# Citreoviridin Enhances Atherogenesis in Hypercholesterolemic ApoE-Deficient Mice via Upregulating Inflammation and Endothelial Dysfunction

**DOI:** 10.1371/journal.pone.0125956

**Published:** 2015-05-01

**Authors:** Hai-Feng Hou, Na Yuan, Qing Guo, Tao Sun, Cheng Li, Jian-Bao Liu, Qun-Wei Li, Bao-Fa Jiang

**Affiliations:** 1 School of Public Health, Taishan Medical University, Taian 271000, China; 2 School of Public Health, Shandong University, Jinan 250012, China; 3 Taian City Central Hospital, Taian 271000, China; 4 Ruijin Hospital, College of Medicine, Shanghai Jiao Tong University, Shanghai 200025, China; Max-Delbrück Center for Molecular Medicine (MDC), GERMANY

## Abstract

Vascular endothelial dysfunction and inflammatory response are early events during initiation and progression of atherosclerosis. *In vitro* studies have described that CIT markedly upregulates expressions of ICAM-1 and VCAM-1 of endothelial cells, which result from NF-κB activation induced by CIT. In order to determine whether it plays a role in atherogenesis *in vivo*, we conducted the study to investigate the effects of CIT on atherosclerotic plaque development and inflammatory response in apolipoprotein E deficient (apoE^-/-^) mice. Five-week-old apoE^-/-^ mice were fed high-fat diets and treated with CIT for 15 weeks, followed by assay of atherosclerotic lesions. Nitric oxide (NO), vascular endothelial growth factor (VEGF) and endothelin-1 (ET-1) were detected in serum. Levels of intercellular adhesion molecule-1 (ICAM-1), vascular cell adhesion molecule-1 (VCAM-1), VEGF, and ET-1 in plaque areas of artery walls were examined. NF-κB p65 expression and NF-κB activation in aorta also were assessed. CIT treatment significantly augmented atherosclerotic plaques and increased expressions of ICAM-1, VCAM-1, VEGF and ET-1 in aorta. Mechanistic studies showed that activation of NF-κB was significantly elevated by CIT treatment, indicating the effect of CIT on atherosclerosis may be regulated by activation of NF-κB.

## Introduction

Atherosclerosis is the primary cause of cardiovascular disease and stroke, which are major causes of morbidity and mortality worldwide [1]. Therefore, it is important to assess the effects of cardiovascular risk factors. Vascular endothelium has major regulatory functions in blood vessel [2]. When the endothelium responds to physiological stress and chemical substances, this results in vascular endothelial dysfunction and an inflammatory process, which leads to the earliest changes that occur during atherosclerosis [3]. Endothelial dysfunction is a significant imbalance of blood-vessel regulating substances produced by the vascular endothelium, such as nitric oxide (NO) and endothelin-1 (ET-1). Moreover, the activation of factor-kappa B (NF-κB) nuclear translocation is known as one of the pathways of inflammatory response, and its action upregulates secretion of inflammatory mediators, such as vascular endothelial growth factor (VEGF), intercellular adhesion molecule-1 (ICAM-1) and vascular cell adhesion molecule-1 (VCAM-1). These play pivotal roles in the early stage of atherosclerosis [4].

Citreoviridin (CIT) is one of the mycotoxins derived from *Penicillium* strains that have been demonstrated to be the pathogenic factor for the acute cardiac beriberi that is prevalent in Japan and Brazil [5–7]. Field investigation indicated that the prevalence rates of cardiovascular disease are high in areas of severe CIT contamination in China, where the contamination levels of CIT in grains are about 5–30 μg/kg [8]. *In vitro* studies illustrate that CIT markedly augments NF-κB activation of endothelial cells, upregulates expressions of ICAM-1 and VCAM-1, and this results in endothelial cell adhesion to monocytes [9]. However, it remains to be known how CIT affects atherogenesis *in vivo*. To determine the effect of CIT on atherosclerosis, we fed 5-week-old apolipoprotein E knockout (apoE^-/-^) mice with high-fat diets, and treated them with CIT for 15 weeks. Our results demonstrate that CIT increased the progress of atherosclerosis in hypercholesterolemic apoE-deficient mice via upregulating inflammation and endothelial dysfunction

## Materials and Methods

### Materials

Citreoviridin (purity≥97% by HPLC) was provided by Fermentek Company (Jerusalem, Israel). Rat anti-ICAM and rat anti-VCAM were obtained from Abcam Biotechnology (Cambridge, UK). Rat anti- ET-1 and rat anti-VEGF were purchased from Santa Cruz Biotechnology (Santa Cruz, CA, USA). ET-1 and VEGF enzyme-linked immunosorbent assay (ELISA) kits were obtained from R&D Systems Inc (Minneapolis, MN, USA). Oil red O was provided by Sigma Chemical Co (St. Louis, MO, USA). Assay kits for total cholesterol (TC), triglyceride (TG) and low-density lipoprotein cholesterol (LDL-C) were purchased from Biosino Biotechnology & Science Inc (Beijing, China). NO assay kit was provided by Nan-jing Jiancheng Bioengineering Co (Nanjing, China). The primary rabbit anti-β-actin, rabbit anti-I-κB, rabbit anti-phospho-I-κB (Ser32), rabbit anti-NF-κB p65 and rabbit anti-phospho-NF-κB p65(Ser536) were obtained from Cell Signaling Technology Inc. (Danvers, MA, USA).

### Ethics Statement

This study was carried out in strict accordance with national guidelines for the care and use of animals. The protocol was approved by the laboratory animals’ ethical committee of Taishan Medical University (Permit Number: 2013–096). All surgery was performed under sodium pentobarbital anesthesia, and all efforts were made to minimize suffering.

### Animals

Five-week-old male mice that were homozygous apoE^-/-^ on C57BL/6J background were provided by Vital River Laboratory Animal Tech. (Beijing, China), and fed with a high-fat diet (Keaoxieli Diet Co., Beijing, China), which contained 21% fat and 0.15% cholesterol. The mice were randomly allocated to three groups, and were administered an oral gavage of 0.5 mg/kg per day CIT, 1 mg/kg per day CIT, or vehicle for 15 weeks. The mice were housed in a specific pathogen-free (SPF) facility with a 12-h light-dark cycle. Health monitoring during and at the end of the study indicated no change in SPF status.

### Collection of Blood and Tissue

After 15 weeks of treatment, the mice were fasted for 12 hours and anesthetized with an intraperitoneal injection of sodium pentobarbital (40 mg/kg). Blood samples were collected from the retro-orbital sinus. The mice were euthanized by exsanguination, and perfusion-fixed with PBS via the left ventricle. The aortas were isolated and stored in liquid nitrogen. The heart and proximal aorta were excised from the aorta at the base, embedded immediately into optimum cutting temperature (OCT) reagent, and frozen in liquid nitrogen.

### Histology and Immunohistochemistry

To quantify the atherosclerotic lesions, the whole aorta, including the ascending arch, thoracic, and abdominal segments, was collected. The adventitia of the aorta was isolated and cut open longitudinally under a dissecting microscope. Five aortas in each group were analyzed using oil red O staining. The percentage of total plaque area to the whole aorta was quantified with Image-Pro-Plus 6.0 software (IPP, Media Cybernetics, MD, USA). To measure atherosclerotic plaques in the aortic root, cryostat sections (8 μm) were prepared as described in the literature [10]. Atherosclerotic plaques in the aortic root were diagnosed at three locations that were separated by a 120 μm distance, and five serial sections were prepared from each location. Then one of the sections in each location was assayed with oil red O staining. The slides were imaged by light microscopy, and the atherosclerotic lesion area was quantified as the percentage of oil red positive area to the total vessel wall area.

The levels of ICAM-1, VCAM-1, VEGF, and ET-1 in plaque areas of the vascular wall were assayed by immunohistochemical analysis. The sections were immersed in 3% hydrogen peroxide(H_2_O_2_) for 15 min to block endogenous peroxidase, and incubated in 5% bovine serum albumin (BSA) buffer to block non-specific binding. The samples were then incubated with primary antibodies overnight at 4°C at the following dilutions: anti-VCAM-1 (1:200), anti-ICAM-1 (1:200), anti-ET-1 (1:400) and anti-VEGF antibodies (1:400). The blank control experiments included omission of the primary antibody. After being washed in PBS, the sections were incubated with goat anti-rat IgG for 30 min at 37°C and detected with 3,3’-diaminobenzidine tetrahydrochloride (DAB) substrate kits (ZSGB-Bio, Beijing, China). All images were captured with an Olympus BX51 microscope equipped with a camera. Brown staining area of image represented target protein expression, and the integrated optical density (IOD) of brown area was calculated by IPP software. The mean IOD value represents the expression level of target protein in the section. In each case, the average value of five locations of each animal was used for analysis.

### Serum Analyses

TC and TG were detected with enzymatic colorimetric assays, and LDL-C was determined according to the Fridewald formula. The serum level of NO was assayed by the nitric acid reductase method. VEGF and ET-1 levels in serum were measured by ELISA kits. All serum parameters were determined by using commercially available kits according to the manufacturer’s instructions.

### Quantitative Real-Time PCR

The qRT-PCR assay was performed to determine the expression levels of ICAM-1 and VCAM-1 mRNA. Total RNA of the aorta was extracted using Trizol reagent (Invitrogen, CA, USA) in accordance with the manufacturer’s protocol. The concentration of RNA was detected by NanoDrop 1100 (NanoDrop Tech., DE, USA). The following primers synthesized by TaKaRa(Dalian, China) were used: GAPDH: forward primer: 5’- CTCATGACCACAGTCCATGC-3’, reverse primer: 5’- CACATTGGGGGTAGGAACAC-3’; VCAM-1: forward primer: ATTTTCTGGGGCAGGAAGTT, reverse primer: ACGTCAGAACAACCGAATCC; ICAM-1 forward 5’- TGCCTCTGAAGCTCGGATATAC-3’, reverse 5’- TCTGTCGAACTCCTCAGTCAC-3’. The cDNAs were synthesized using MuLV Reverse Transcriptase (Applied Biosystems, CA, USA). The qRT-PCR was performed with a Rotor-Gene Q real-time PCR cycler (Qiagen, Germany) using a SYBR-green PCR master mix kit (Applied Biosystems, CA, USA). Blank controls (omission of template or RNA) were designed to make sure of the absence of contamination. Data were analyzed using Rotor-gene Q v1.7 (Qiagen, Germany), and relative mRNA levels were quantified by the 2^−ΔΔCt^ method and normalized by GAPDH mRNA. Each experiment was repeated five times.

### Western Blotting

Five aortas in each group were collected and analyzed by Western blotting. The trituration of aorta samples were lysed at 4°C for 1 h in lysis buffer (150 mM NaCl, 20 mM Tris-HCl, pH 7.4, 0.1% SDS, 1.0% NP-40, 0.5% Na-deoxycholate, 0.2 mM PMSF, and protease inhibitor cocktails). Then the lysates were centrifuged at 12,000*g* for 20 min. The lysates (about 20 μg protein) were separated in 10%SDS-PAGE gels, and transferred to polyvinylidene fluoride (PVDF) membranes, blocked with 8% nonfat milk powder for 60 min, and probed with the relevant primary antibodies at 4°C overnight. The blots were incubated with horseradish peroxidase (HRP)-conjugated anti-IgG for 1h at 37°C, and detected with the ECL Western Blotting Detection Reagents (Thermo Scientific Pierce, Rockford, IL, USA).

### Statistical Analysis

All data are presented as means ± standard error of mean (SEM) for 10 animals per group. Statistical significance analysis was determined by applying one-way analysis of variance (ANOVA) and the Student-Newman-Keuls (SNK) multiple comparison test. Statistical analysis was carried our with SPSS software (v 20.0, SPSS Inc. Chicago, USA). Probability values less than 0.05 were considered statistically significant.

## Results

### Body Weight and Serum Lipid Levels

**Fig 1:**
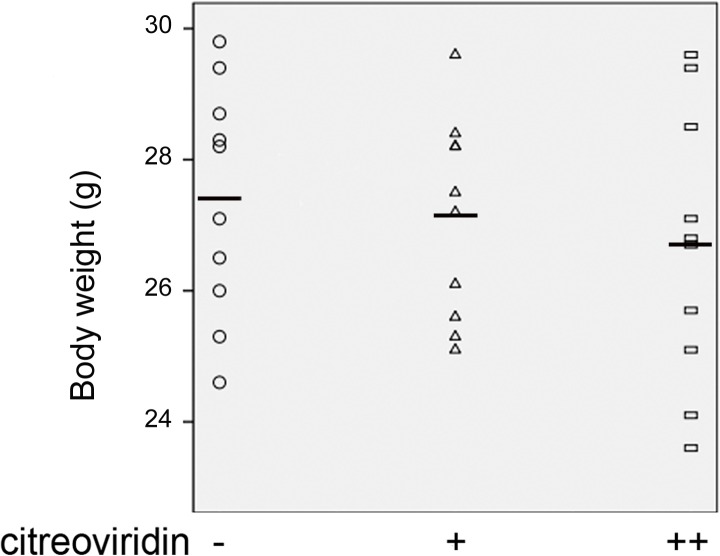
Body weight of apoE^-/-^ mice. Data are presented as mean ± SEM (n = 10). The statistical analysis of one-way analysis of variance (ANOVA) was performed. There was no significant difference among the three groups.

**Fig 2:**
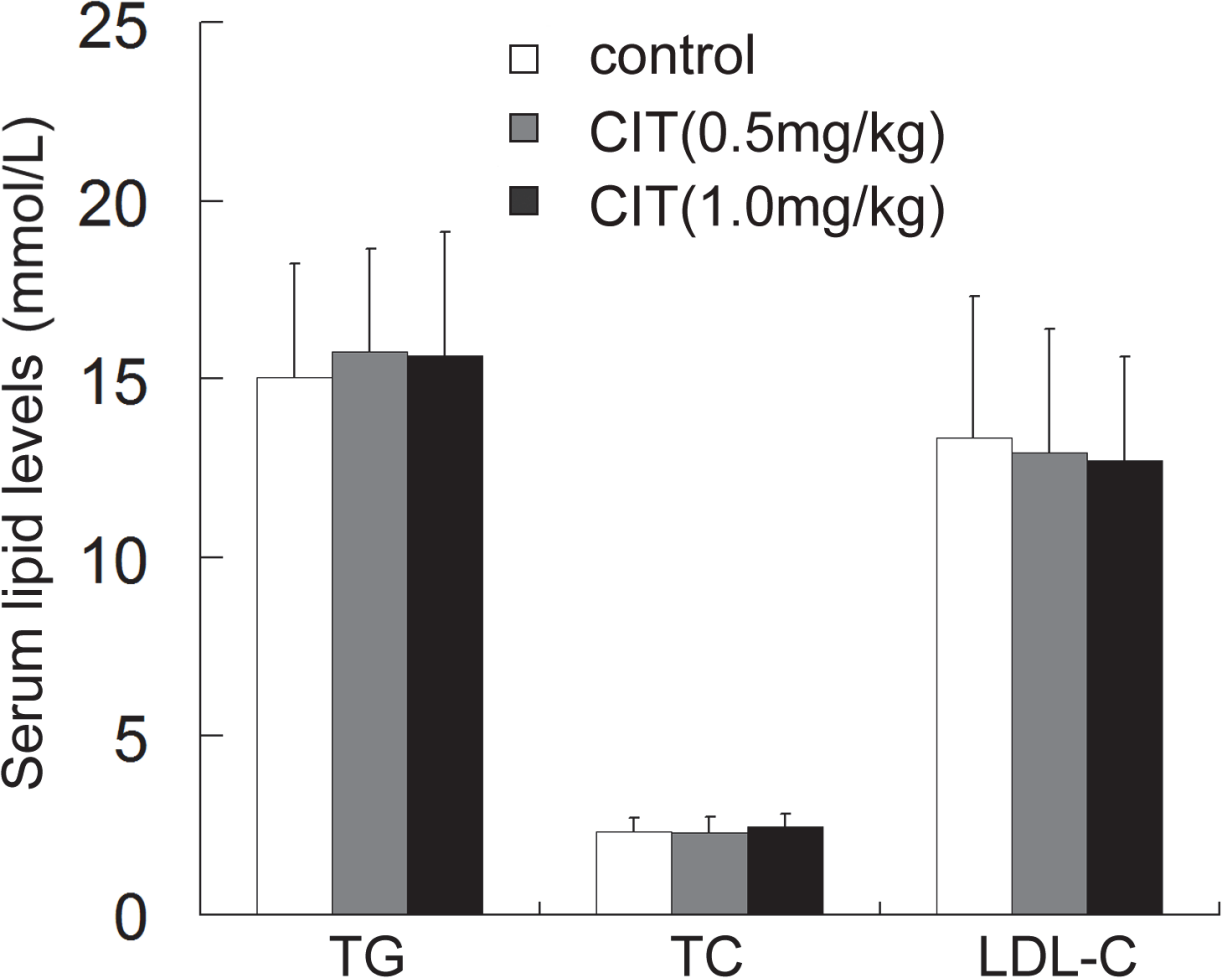
Serum lipid levels in apoE^-/-^ mice. Data are presented as mean ± SD (n = 10). The statistical analysis of one-way analysis of variance (ANOVA) was performed. There was no significant difference among the three groups.

Body weight gain and hyperlipidemia of apoE^-/-^ mice were induced by high-fat diets for 15 weeks. As shown in Fig 1, the final weights of mice in the control group, 0.5 mg/kg CIT group and 1 mg/kg CIT group were 27.39±1.76 g, 27.12±1.53 g, and 26.66±2.08 g, respectively. No significant effects of CIT on body weight were observed. Moreover, results presented in Fig 2 showed that CIT treatment did not affect serum TC, TG or LDL-C levels compared to mice in the control group treated with vehicle. The relevant data are also presented in S1 Dataset.

### Atherosclerotic Lesions in the Aorta

**Fig 3:**
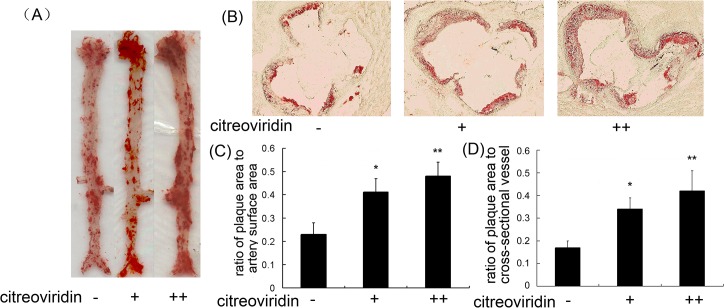
Effect of CIT on atherosclerotic lesions in apoE^-/-^ mice. (A) Representative image of en face Oil Red O staining of aorta. (B) Representative cross-sectional view of aortic root stained with oil red O (40×). (C) Ratio of plaque area stained with oil red O to total luminal surface area. Data are presented as mean ± SEM (n = 5). (D) Plaque area stained with oil red O in aortic root (n = 10). The statistical analyses of one-way analysis of variance (ANOVA) and Student-Newman-Keuls (SNK) multiple comparison were performed to determine the significant difference among the three groups. *P < 0.05, **P < 0.01 versus vehicle-treated group; ^**#**^ *P* < 0.05, ^**##**^ *P* < 0.01 versus low dosage CIT treatment group.

As shown in Fig 3, atherosclerotic lesions were visualized in the aorta by oil red O staining. The size of aortic plaques in CIT treatment groups were greater than that in control group, and plaques in the aortic arch and aortic crotch were thicker in CIT groups compared with the control group. The percentage of aortic lesion areas was calculated by IPP software. Compared to the control group, 0.5 mg/kg per day CIT treatment increased the number of aortic plaque areas by 78.2%, and the mice in 1 mg/kg per day CIT treatment group had almost twice as many atherosclerotic plaques compared with the control group. Atherosclerotic lesions also were measured as aortic root cross-sectional lesion areas stained with oil red O. Microscopic analysis showed that CIT treatment augmented atherosclerotic plaques compared to the vehicle-treated group. The results indicate that CIT contributed to atheroma formation in hypercholesterolemic apoE^-/-^ mice.

### NO, ET-1 and VEGF Expression

**Fig 4:**
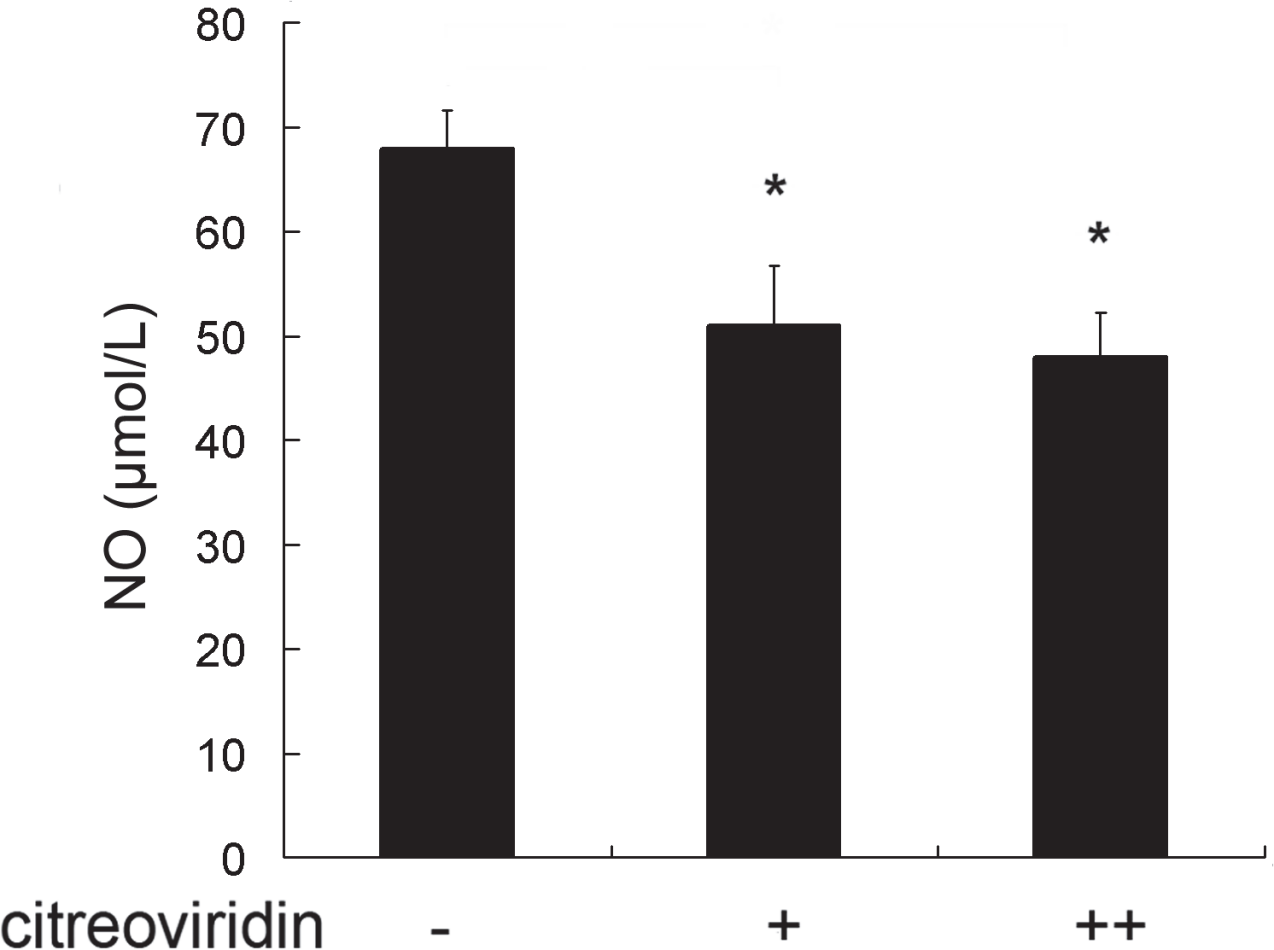
Serum NO concentration in apoE^-/-^ mice. Data are presented as mean ± SEM (n = 10). The statistical analyses of one-way analysis of variance (ANOVA) and Student-Newman-Keuls (SNK) multiple comparison were performed to determine the significant difference among the three groups. *P < 0.05, **P < 0.01 versus vehicle-treated group; ^**#**^ *P* < 0.05, ^**##**^ *P* < 0.01 versus low dosage CIT treatment group.

**Fig 5:**
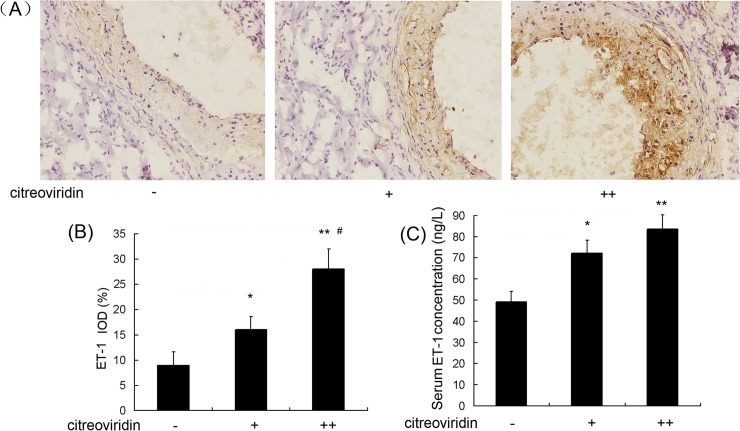
ET-1 expression in aorta of apoE^-/-^ mice treated with CIT. (A) Immunohistochemical staining of aortic sections (blue = nuclei, brown = target protein, 100×) (B) Integral optical density (IOD) values of ET-1. Data are presented as mean ± SEM (n = 10). (C) The concentration of ET-1 in the serum of apoE^**-/-**^ mice (n = 10). The statistical analyses of one-way analysis of variance (ANOVA) and Student-Newman-Keuls (SNK) multiple comparison were performed to determine the significant difference among the three groups. *P < 0.05, **P < 0.01 versus vehicle-treated group; ^**#**^ *P* < 0.05, ^**##**^ *P* < 0.01 versus low dosage CIT treatment group.

**Fig 6:**
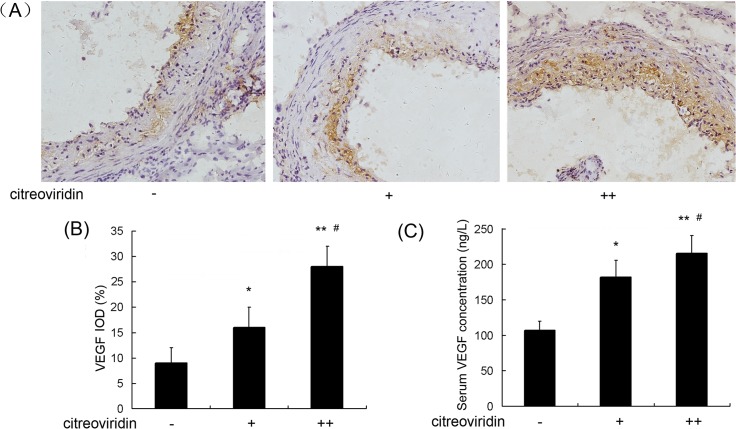
VEGF expression in aorta of apoE^-/-^ mice treated with CIT. (A) Immunohistochemical staining of aortic sections (blue = nuclei, brown = target protein, 100×). (B) Integral optical density (IOD) values of VEGF. Data are presented as mean ± SEM (n = 10). (C) The concentrations of VEGF in serum of apoE^**-/-**^ mice(n = 10). The statistical analyses of one-way analysis of variance (ANOVA) and Student-Newman-Keuls (SNK) multiple comparison were performed to determine the significant difference among the three groups. *P < 0.05, **P < 0.01 versus vehicle-treated group; ^**#**^ *P* < 0.05, ^**##**^ *P* < 0.01 versus low dosage CIT treatment group.

Hypercholesterolemia induces oxidative stress, and upregulates the expression of VEGF in the vessel wall, which has been suggested play a role in the development of atherosclerosis[11]. NO and ET-1 are the main vasodilator and vasoconstrictor substances involved in blood vessel regulation, and are an indicator molecules in the formation of atherosclerosis [12]. As shown in Fig 4, CIT treatment reduced NO levels in low dosage and high dosage CIT treatment groups by 25.4% and 29.1%, respectively, compared with the control group. As shown in Fig 5, the level of serum ET-1 in the 0.5 mg/kg per day CIT group was 45.4% higher than in the control group, and the level in the 1 mg/kg per day CIT group was 66.3% higher than in the control group. Furthermore, positive areas of ET-1 in aortic roots increased by 74.2% and 199.0%, respectively, in the two CIT-treated groups compared to the vehicle-treated group. The concentrations of VEGF in serum and positive areas of VEGF in aortic roots were largely increased in CIT-treated groups (Fig 6).

### CIT Increased ICAM-1 and VCAM-1 Expression

**Fig 7:**
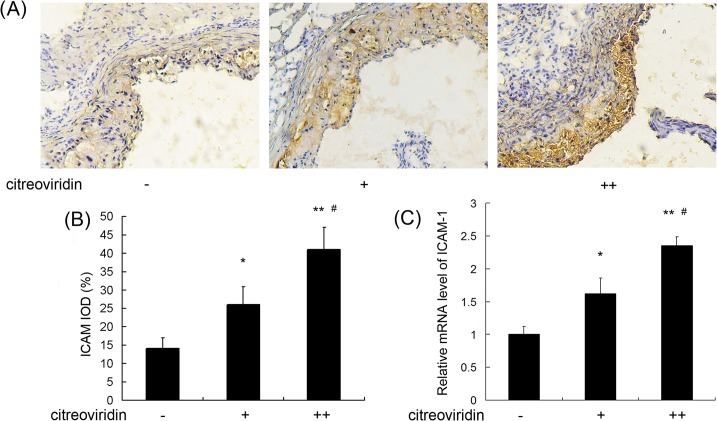
ICAM-1 expression in aorta of apoE^-/-^ mice. (A) Immunohistochemical staining of aortic sections (blue = nuclei, brown = target protein, 100×). (B) Integral optical density (IOD) value of ICAM-1. Data are presented as mean ± SEM (n = 10). (C) The relative ICAM-1mRNA level (n = 5). The values are shown as ratios compared to the levels of mRNA expression in control mice. The statistical analyses of one-way analysis of variance (ANOVA) and Student-Newman-Keuls (SNK) multiple comparison were performed to determine the significant difference among the three groups. *P < 0.05, **P < 0.01 versus vehicle-treated group; ^**#**^ *P* < 0.05, ^**##**^ *P* < 0.01 versus low dosage CIT treatment group.

**Fig 8:**
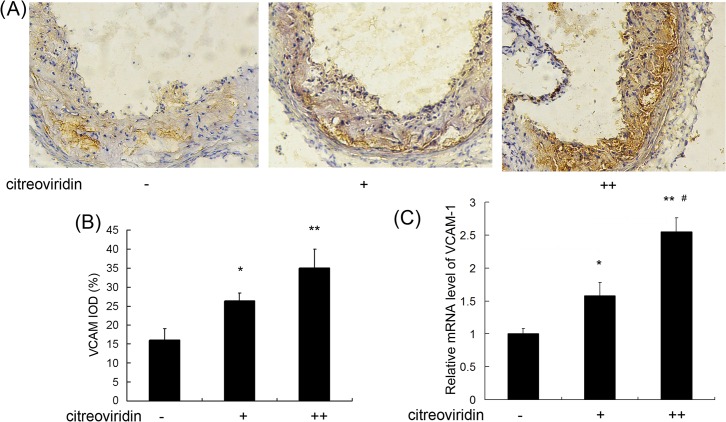
VCAM-1 expression in aorta of apoE^-/-^ mice treated with CIT. (A) Immunohistochemical staining of aortic sections (blue = nuclei, brown = target protein, 100×). (B) Integral optical density (IOD) value of VCAM-1. Data are presented as mean ± SEM (n = 10). (C) The relative VCAM-1mRNA level (n = 5). The values are shown as ratios compared to the levels of mRNA expression in control mice. The statistical analyses of one-way analysis of variance (ANOVA) and Student-Newman-Keuls (SNK) multiple comparison were performed to determine the significant difference among the three groups. *P < 0.05, **P < 0.01 versus vehicle-treated group; ^**#**^ *P* < 0.05, ^**##**^ *P* < 0.01 versus low dosage CIT treatment group.

Cellular adhesion molecules, including ICAM-1 and VCAM-1, are over-expressed by the vascular endothelium in the initial process of atherosclerosis, which may lead to upregulation of endothelium cell adhesion and atherosclerotic lesions. Immunohistochemistry assay showed (Fig 7) CIT treatment increased ICAM-1 levels by 85.8% and 188.7%, respectively, in the 0.5 mg/kg per day and 1 mg/kg per day CIT treatment groups compared with the control group. Furthermore, An increase of ICAM-1 mRNA expression was measured in CIT treatment groups. As shown in Fig 8, CIT upregulated VCAM-1 expression by 65.6% and 116.1%, respectively, in the two CIT treatment groups compared with the control group. The VCAM-1 mRNA levels in CIT treatment groups were also upregulated significantly.

### CIT Increased NF-κB Activation in ApoE^-/-^ Mice Fed High-fat Diets

**Fig 9:**
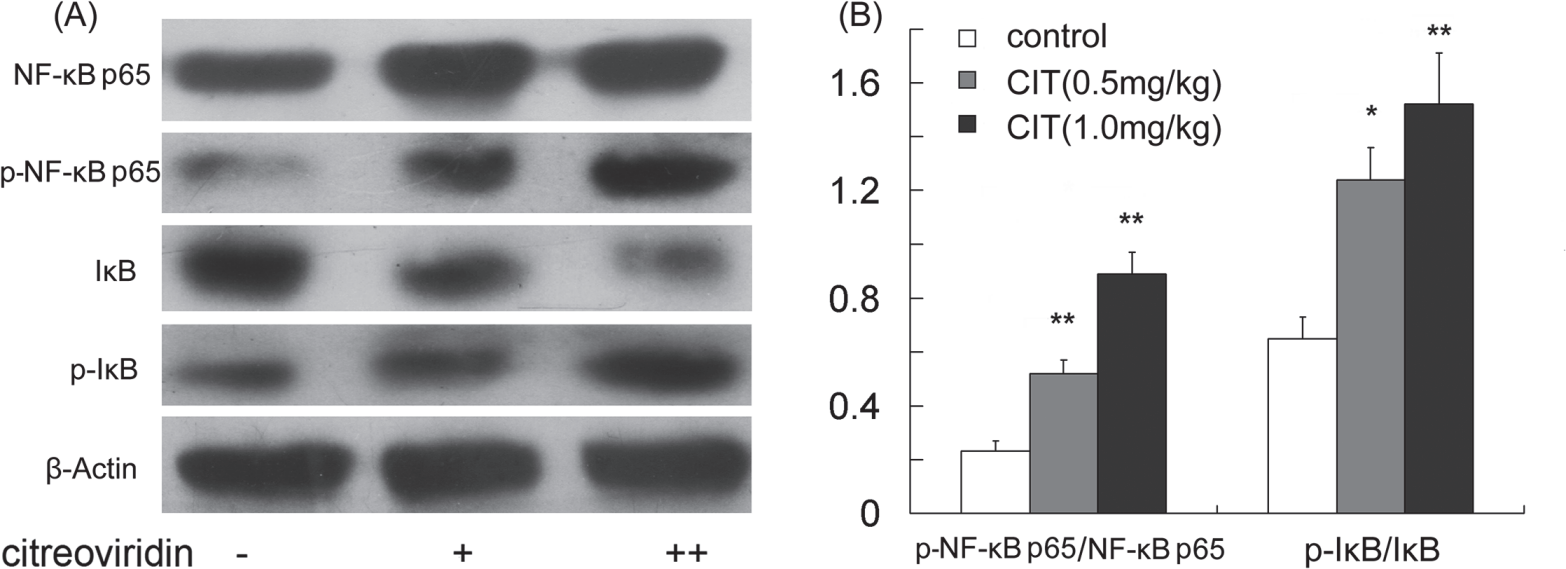
CIT increased NF-κB activation in aorta. (A) Expression of NF-κB p65, phospho-NF-κB p65, I-κB, and phospho-I-κB in aorta analyzed by Western blotting. (B) The levels of phospho-NF-κB p65/NF-κB p65 and phospho-I-κB/I-κB. Data are presented as mean ± SEM (n = 5). The statistical analyses of one-way analysis of variance (ANOVA) and Student-Newman-Keuls (SNK) multiple comparison were performed to determine the significant difference among the three groups. *P < 0.05, **P < 0.01 versus vehicle-treated group; ^**#**^ *P* < 0.05, ^**##**^ *P* < 0.01 versus low dosage CIT treatment group.

We evaluated NF-κB activation by detecting I-κB degradation and NF-κB p65 phosphorylation. As shown in Fig 9, treatment with CIT effectively increased the ratio of p-I-κB/I-κB and the odd of p-NF-κB p65/NF-κB p65. The data indicated that in CIT treatment groups IκB phosphorylation and degradation were increased significantly, which resulted in the augment of NF-κB p65 phosphorylation and nuclear translocation. It is suggested that CIT may contribute to activating NF-κB in vascular endothelium.

## Discussion

CIT is a mycotoxin isolated from several *Penicillium* species, and is mainly produced by *Penicillium citreoviride* and *Penicillium citreonigrum*. CIT is thought to be the toxin responsible for acute cardiac beriberi, which was prevalent in Japan and Brazil, and was responsible for the prevalence of Keshan disease in China, based on early literature [7, 13]. However, not all studies have made this presumption [14]. A recent study illuminated a novel discovery that CIT upregulated adhesion molecules on endothelial cells, which ultimately led to coronary heart disease [9]. The prevalence of cardiovascular disease is high in areas of severe CIT contamination [8]. In this study, we used the apoE^-/-^ mouse atherosclerotic model to evaluate the effects of CIT on vascular endothelium *in vivo*, and clarified the role of CIT in atheroma formation in apoE^-/-^ mice.

The main finding of this study is that CIT promotes atheroma formation. The effects of CIT were linked with the strong upregulation of aortic inflammation (ICAM-1 and VCAM-1) and endothelial dysfunction in the atherosclerotic model. Furthermore, the molecular mechanism of the effects may be the capacity of CIT to promote activation of NF-κB. In addition, CIT enhanced atherosclerotic lesions without significantly altering body weight, or total cholesterol and lipoprotein levels.

Unlike other studies that employed a much higher dose of CIT (5, 10 or 300 mg/kg per day) [15, 16], the dose in the present study was substantially lower, based on the level of CIT contamination in grains[8]. Consequently, CIT did not cause acute toxic myocardial damage, which was observed in earlier studies using a high dosage of CIT [15, 16].

The endothelium regulates vasodilation and vasoconstriction by producing important endogenous mediators, such as nitric oxide (NO) and endothelin (ET) [17]. In the regulation of vascular homeostasis, NO mediates endothelium-dependent vasodilation, while other substances such as ET-1, control the effects of vasoconstrictors [18,19]. When vasomotor function is impaired, this results in endothelial dysfunction, which leads to the initial changes of atherosclerosis[20]. We assessed the effect of CIT on endothelial dysfunction in the atherosclerotic animal model by measuring the levels of NO and ET in serum. The results showed that CIT aggravated the imbalance of serum NO and ET-1, which was a key indicator of endothelial dysfunction. Consequently, the atherosclerotic plaque areas were augmented by CIT, and atherosclerotic lesions were more serious in mice treated with the high dosage of CIT than in the low-dose group.

Adhesion molecules (*e*.*g*. ICAM-1 and VCAM-1) released by vascular endothelium have been identified as another activators in the earlier phases of atherosclerosis [21]. ICAM-1 and VCAM-1 modify the process underlying the adhesion between endothelial cells and monocytes, which leads mononuclear cells into artery intima and results in the early lesions of atherosclerosis [21–23]. In this study, we showed that CIT upregulated the expression levels of ICAM-1 and VCAM-1 in the apoE^-/-^ mice. In addition, increases in atherosclerotic plaque were accompanied by expression of ICAM-1 and VCAM-1.

VEGF is an important chemotactic factor during vascular inflammation [11, 24]. Increased VEGF expression has been demonstrated in hypercholesterolemic animal models, and VEGF protein has high-level expression among patients with hyperlipidemia and atherosclerosis [25, 26]. In this study, we found that CIT treatment increased levels of VEGF in the aorta specimens and serum. The results showed that CIT upregulated VEGF expression.

Recently, CIT has been identified to be an assistant activator of NF-κB in TNFα induced endothelial cell adhesion[9]. Nuclear translocation of the cytosolic NF-κB p65 subunit in vascular endothelial cells, known as NF-κB activation, is an important pathway in the development of atherosclerosis [27]. The function of NF-κB is restricted to the cytoplasm by binding to the inhibitor IκB. The phosphorylation of IκB induces its degradation, and results in NF-κB p65 translocation into the nucleus where phosphor-NF-κB promotes the expression of cytokines [28,29]. This pathway contributes to the upregulation of pro-inflammatory mediators, such as ICAM-1 and VCAM-1 [30]. In present study, CIT treatment promoted IκB phosphorylation and degradation, which consequently caused NF-κB p65 phosphorylation and activation. The expression of phospho-NF-κB p65 was consistent with that of ICAM-1 and VCAM-1. Therefore, NF-κB activation may play a pivotal role in contributing to the endothelial inflammatory process modulated by CIT. Our findings *in vivo* are similar to the research evidence *in vitro*[9].

In summary, CIT enhances atherogenesis in hypercholesterolemic apoE-deficient mice via upregulating inflammation and endothelial dysfunction. CIT may well be a risk factor for vascular disease. Further studies are needed to identify the mechanisms by which CIT regulate NF-κB activation.

## Supporting Information

S1 Dataset The original data of the article. (XLS)
